# Comparative Genomics of *Helicobacter pylori *and the human-derived *Helicobacter bizzozeronii *CIII-1 strain reveal the molecular basis of the zoonotic nature of non-pylori gastric *Helicobacter *infections in humans

**DOI:** 10.1186/1471-2164-12-534

**Published:** 2011-10-31

**Authors:** Thomas Schott, Pradeep K Kondadi, Marja-Liisa Hänninen, Mirko Rossi

**Affiliations:** 1 Department of Food Hygiene and Environmental Health (DFHEH), Faculty of Veterinary Medicine, University of Helsinki, P.O. Box 66, FI-00014 University of Helsinki, Finland

## Abstract

**Background:**

The canine Gram-negative *Helicobacter bizzozeronii *is one of seven species in *Helicobacter heilmannii *sensu lato that are detected in 0.17-2.3% of the gastric biopsies of human patients with gastric symptoms. At the present, *H. bizzozeronii *is the only non-pylori gastric *Helicobacter *sp. cultivated from human patients and is therefore a good alternative model of human gastric *Helicobacter *disease. We recently sequenced the genome of the *H. bizzozeronii *human strain CIII-1, isolated in 2008 from a 47-year old Finnish woman suffering from severe dyspeptic symptoms. In this study, we performed a detailed comparative genome analysis with *H. pylori*, providing new insights into non-pylori *Helicobacter *infections and the mechanisms of transmission between the primary animal host and humans.

**Results:**

*H. bizzozeronii *possesses all the genes necessary for its specialised life in the stomach. However, *H. bizzozeronii *differs from *H. pylori *by having a wider metabolic flexibility in terms of its energy sources and electron transport chain. Moreover, *H. bizzozeronii *harbours a higher number of methyl-accepting chemotaxis proteins, allowing it to respond to a wider spectrum of environmental signals. In this study, *H. bizzozeronii *has been shown to have high level of genome plasticity. We were able to identify a total of 43 contingency genes, 5 insertion sequences (ISs), 22 mini-IS elements, 1 genomic island and a putative prophage. Although *H. bizzozeronii *lacks homologues of some of the major *H. pylori *virulence genes, other candidate virulence factors are present. In particular, we identified a polysaccharide lyase (HBZC1_15820) as a potential new virulence factor of *H. bizzozeronii*.

**Conclusions:**

The comparative genome analysis performed in this study increased the knowledge of the biology of gastric *Helicobacter *species. In particular, we propose the hypothesis that the high metabolic versatility and the ability to react to a range of environmental signals, factors which differentiate *H. bizzozeronii *as well as *H. felis *and *H. suis *from *H. pylori*, are the molecular basis of the of the zoonotic nature of *H. heilmannii *sensu lato infection in humans.

## Background

*Helicobacter pylori *is established as the primary cause of gastritis and peptic ulceration in humans and has been recognised as a major risk factor for mucosa-associated lymphoid tissue (MALT) lymphoma and gastric adenocarcinoma [1]. However, in gastric biopsies of a minority of patients (0.17-2.3%) with upper gastrointestinal symptoms, long, tightly coiled spiral bacteria, referred to non-*H. pylori *gastric *Helicobacter *species (NHPGH), have been observed [2,3]. Although NHPGH-associated gastritis is usually mild to moderate compared to *H. pylori *gastritis, severe complications of NHPGH infection have been reported, and it is important to note that MALT lymphoma is significantly more frequent in NHPGH-infected patients compared to those infected with *H. pylori *[4].

As a strictly human-associated pathogen, *H. pylori *is transmitted from human to human. In contrast, NHPGH are zoonotic agents for which animals are the natural reservoir [5]. Recently, the term *H. heilmannii *sensu lato has been proposed to be used to refer to the whole group of NHPGH detected in the human or animal stomach when only morphology and limited taxonomical data are available [3]. Indeed, *H. heilmannii *s.l. comprises seven described *Helicobacter *species: the porcine *Helicobacter suis*; the feline *Helicobacter felis*, *Helicobacter baculiformis *and *Helicobacter heilmannii *sensu stricto (s.s.); and the canine *Helicobacter bizzozeronii*, *Helicobacter salomonis *and *Helicobacter cynogastricus *[3]. These *Helicobacter *species are very fastidious microorganisms and difficult to distinguish from each others with conventional identification procedures. Due to difficulties in the isolation and identification of *H. heilmannii *s.l., its epidemiology in human infections remains unclear. Although development of specific isolation methods has allowed to identify several new species considered to be uncultivable (i.e.: *H. suis *and *H. heilmannii *s.s. [2,3]), up to now only *H. bizzozeronii *has been cultivated from the gastric mucosa of two human patients [6-8].

*H. bizzozeronii*, described as a new species in 1996 [9], is a canine-adapted species that is often found during a histological examination of gastric biopsies of dogs both with and without morphological signs of gastritis [10]. Virtually all animals are infected, and the pathogenic significance of *H. bizzozeronii *in dogs remains unknown [2,10]. Experimental infection studies using mice [11] and gerbils [12] have suggested that *H. bizzozeronii *appears to be associated with a lower pathogenicity than *H. pylori *or *H. felis*. Even in cases of heavy bacterial loading, the only pathological finding observed was focal apoptotic loss of gastric epithelial cells [12]. Despite the lack of connection between *H. bizzozeronii *and gastric diseases in both naturally and experimentally-infected animals, *H. bizzozeronii *has been associated in both human cases with severe dyspeptic symptoms [7,8]. Moreover, the human *H. bizzozeronii *strain CIII-1 was isolated from antrum and corpus biopsies that revealed chronic active gastritis [8], indicating that this species is able to induce similar injuries in the human gastric mucosa to those observed in *H. pylori*-infected patients.

Several genome sequences of *H. pylori *are available, and recently, the genomes of other gastric *Helicobacter *species were also published [13-16]. Among *H. heilmannii *s.l., the genomes of the type strain of *H. felis *[16], isolated from a cat, and of two strains of *H. suis *[15], both isolated from pigs, were determined. Recently, we sequenced the genome of the *H. bizzozeronii *human strain CIII-1, isolated in 2008 from a 47-year-old Finnish woman suffering from severe dyspeptic symptoms [17]. To our knowledge, *H. bizzozeronii *CIII-1 is the only human *H. heilmannii *s.l. strain for which the genome is available and constitutes a keystone in the comprehension of non-pylori *Helicobacter *infections and of the mechanisms of transmission between the primary animal host and humans. Moreover, although the role of *H. bizzozeronii *in human gastric disease is limited compared to *H. pylori*, both of the species persistently colonise the same niche and possibly exploit similar mechanisms to interact with the host and induce gastritis. Thus, a comparative genome analysis between *H. pylori *and the human *H. bizzozeronii *CIII-1 would expand our knowledge of the biology of gastric *Helicobacter *spp. and of the pathogenesis of human gastritis.

In this study, we provide a detailed comparative genome analysis of *H. pylori *and the human *H. bizzozeronii *CIII-1 strain. We advance the hypothesis that the zoonotic nature of *H. bizzozeronii*, and, by extension of *H. heilmannii *s.l., is explicable from its high metabolic versatility, which probably supports the growth of this bacterium in a wide range of niches.

## Results and discussion

### General features and comparison with other taxa

**Figure 1:**
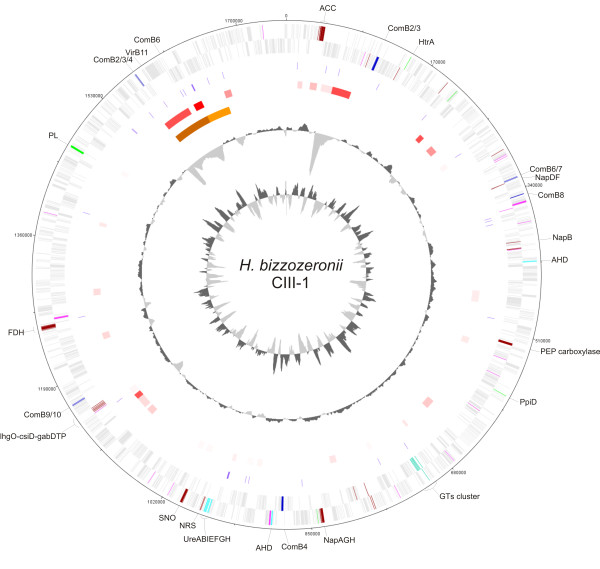
**Circular representation of the *Helicobacter bizzozeronii *CIII-1 chromosome**. From the outside in: the outer circle 1 shows the size in base pairs; circles 2 and 3 show the position of CDS transcribed in a clockwise and anti-clockwise direction, respectively (for color codes see below); circle 4 shows the position of insertion elements (IS and mini-IS, violet); circle 5 shows the results from Alien Hunter program from the lowest score (light pink) to the highest score (red) (threshold score value: 16.971); circle 6 shows the positions of the genomic island (brown) and prophage (orange); circle 7 shows a plot of G+C content (in a 10-kb window); circle 8 shows a plot of GC skew ([G_C]/[G+C]; in a 10-kb window). Genes in circles 2 and 3 are color-coded according to the following category: wine red, genes involved in central metabolism and respiration without orthologues in *H. pylori*; cyan, methyl-accepting chemotaxis proteins (MCPs); dark blue, type IV secretion system; sky blue, genes involved in acid acclimation; green, putative secreted virulence factors; pale green, glycosyltransferse gene cluster specific of *H. bizzozeronii*; pale grey, all other CDSs. ACC, acetophenone carboxylase; comB, Type IV secretion system; NAP, periplasmic nitrate reductase; AHD, allophanate hydrolase; GT, glycosyltransferase; NRS, nitrite reductase system; SNO, S- and N- oxidases; FDH, formate reductase system; PL, polysaccharide lyase.

The general features of the *H. bizzozeronii *CIII-1 genome were previously described [17]. Briefly, the chromosome is similar in size and GC content (46%) to other sequenced gastric *Helicobacter *species [13,16] and includes 1,894 protein-coding sequences (CDSs) in a coding area of 93%. A putative function could be predicted for 1,280 (67.7%) of the CDSs, whereas 614 (32.4%) of the CDSs were annotated as hypothetical proteins. A summary of the features of the *H. bizzozeronii *CIII-1 genome is provided in Table 1, and a circular plot of the chromosome showing GC content and GC skew is presented in Figure 1.

**Table 1:** Features of the *Helicobacter bizzozeronii *CIII-1 genome

	Number or % of total
General features	
Chromosome size (bp)	1,755,458
G+C content	46%
CDS numbers	1,894
Assigned function	1,280
Conserved hypothetical/hypothetical protein	614
Ribosomal RNA operons	2
tRNAs	36
Prophage	1
Genetic island	1
Number of plasmids (bp)	1 (52,076)
IS elements	5
mini-IS elements	22
Simple sequence repeats (SSRs)	80
mono ≥ 9	71
di ≥ 6	6
tetra ≥ 4	0
penta ≥ 4	2
hepta ≥ 4	1
RAST Subsystem Category Distribution*	
Cofactors, Vitamins, Prosthetic Groups, Pigments	7.9%
Cell Wall and Capsule	8.2%
Virulence, Disease and Defense	1.6%
Potassium metabolism	0.8%
Miscellaneous	2.8%
Membrane Transport	2.5%
Iron acquisition and metabolism	0.3%
RNA Metabolism	6.4%
Nucleosides and Nucleotides	4.4%
Protein Metabolism	18.1%
Cell Division and Cell Cycle	1.7%
Motility and Chemotaxis	5.9%
Regulation and Cell signaling	0.3%
DNA Metabolism	3.0%
Fatty Acids, Lipids, and Isoprenoids	5.7%
Respiration	5.9%
Stress Response	2.4%
Metabolism of Aromatic Compounds	0.6%
Amino Acids and Derivatives	11.6%
Sulfur Metabolism	0.3%
Phosphorus Metabolism	0.6%
Carbohydrates	8.8%
Gene classes	
Chemotaxis proteins	23
HAMP domain containg protein	8
Methyl-accepting chemotaxis proteins (MCPs)	20
HD domain containg protein	2
Redox-sensing PAS domain proteins	1
Restriction/modification systems	
Type I	1(on plasmid)
Type II/IIS	2
Type III	1(partial)
Transcriptional regulators	3(σ^28 ^σ^54 ^σ^70^)
Two-component systems	
Response regulator	10
Sensor histidine kinase	4

*percentage of 1,151 CDS included in 234 subsystems by RAST server (http://rast.nmpdr.org/)

The taxonomy of the best BLAST hits for the *H. bizzozeronii *CIII-1 CDSs is shown in Table 2. Approximately 94% of the CIII-1 proteins have their best matches in proteins encoded by ε-proteobacteria, 97% of which belong to *Helicobacteracae*, and 95% are similar to proteobacterial proteins. *H. felis *is the most closely related organism, covering almost 50% of the best matches, followed by *H. suis *(36%) and *H. pylori *(2.7%). However, 33% of the CDSs have a homologue from *H. pylori *within the 5 best BLAST matches. Among the *Campylobacter *spp., *C. rectus *is the closest to *H. bizzozeronii *(0.9%; 16/1894) even though the homology is limited to the prophage region (see below). Matches to non-ε-proteobacteria are found within the Firmicutes (1.58%), γ-proteobacteria (0.9%), Bacteroides (0.6%) and also within the Eukaryota (0.9%).

**Table 2:** Similarity of predicted *Helicobacter bizzozeronii *CIII-1 proteins to proteins from other taxa

Taxon	Best match		Within best 5 matches
	#	%	%
*Proteobacteria*	1,817	95.53	91.55
Epsilon	1,791	94.16	87.55
*Helicobacteraceae*	1,733	91.11	81.74
*Helicobacter felis*	937	49.26	19.09
*Helicobacter suis*	690	36.28	17.10
*Helicobacter pylori*	51	2.68	33.04
*Helicobacter mustelae*	12	0.63	1.90
*Helicobacter cinaedi*	8	0.42	1.67
*Helicobacter hepaticus*	8	0.42	1.67
*Helicobacter bilis*	8	0.42	1.25
*Helicobacter acinonychis str. Sheeba*	5	0.26	2.92
*Helicobacter winghamensis*	5	0.26	0.46
*Candidatus Helicobacter heilmannii*	3	0.16	0.06
*Wolinella succinogenes*	2	0.11	0.79
*Helicobacter cetorum*	2	0.11	0.17
*Helicobacter canadensis*	1	0.05	0.63
*Helicobacter pullorum*	1	0.05	0.63
*Sulfuricurvum kujiense*	0	0.00	0.16
*Sulfurimonas autotrophica*	0	0.00	0.09
*Sulfurimonas denitrificans*	0	0.00	0.08
*Helicobacter salomonis*	0	0.00	0.01
*Campylobacteraceae*	54	2.84	5.18
*Campylobacter rectus*	16	0.84	0.38
*Campylobacter jejuni*	11	0.58	1.49
*Campylobacter upsaliensis*	9	0.47	0.77
*Campylobacter concisus*	6	0.32	0.24
*Campylobacter coli*	5	0.26	0.28
*Campylobacter fetus*	4	0.21	0.41
*Campylobacter curvus*	2	0.11	0.29
*Campylobacter gracilis*	1	0.05	0.12
*Sulfurospirillum sp*.	0	0.00	0.30
*Arcobacter butzleri*	0	0.00	0.27
*Arcobacter nitrofigilis*	0	0.00	0.21
*Campylobacter showae*	0	0.00	0.20
*Campylobacter lari*	0	0.00	0.18
*Campylobacter hominis*	0	0.00	0.03
Unclassified	3	0.16	0.39
*Nautiliaceae*	1	0.05	0.24
Alpha	4	0.21	0.49
Beta	4	0.21	0.78
*Burkholderiales*	4	0.21	0.58
Others	0	0.00	0.20
Gamma	18	0.95	2.50
*Aeromonadales*	5	0.26	0.26
*Pasteurellales*	4	0.21	0.47
*Enterobacteriales*	2	0.11	0.45
*Pseudomonadales*	1	0.05	0.39
*Cardiobacteriales*	1	0.05	0.25
*Vibrionales*	1	0.05	0.16
*Xanthomonadales*	1	0.05	0.14
*Oceanospirillales*	1	0.05	0.11
*Chromatiales*	1	0.05	0.05
*Thiotrichales*	0	0.00	0.08
*Legionellales*	0	0.00	0.02
Others	1	0.05	0.12
Delta	0	0.00	0.22
*Firmicutes*	30	1.58	2.80
*Clostridiales*	15	0.79	1.34
*Bacillales*	11	0.58	0.52
*Lactobacillales*	2	0.11	0.48
*Selenomonadales*	1	0.05	0.24
*Erysipelotrichales*	1	0.05	0.16
*Thermoanaerobacterales*	0	0.00	0.06
*Bacteroidetes/Chlorobi*	12	0.63	1.01
*Bacteroidales*	5	0.26	0.42
*Flavobacteriales*	3	0.16	0.25
*Sphingobacteriales*	2	0.11	0.08
Unclassified	1	0.05	0.16
*Chlorobiales*	1	0.05	0.03
*Cytophagales*	0	0.00	0.06
*Spirochaetes*	7	0.37	0.77
*Chloroflexi*	3	0.16	0.08
*Fusobacteria*	2	0.11	0.59
*Archaea*	2	0.11	0.18
*Actinobacteria*	1	0.05	0.37
*Fibrobacteres/Acidobacteria*	1	0.05	0.16
*Cyanobacteria*	1	0.05	0.13
*Aquificae*	1	0.05	0.05
*Deferribacteres*	1	0.05	0.04
*Tenericutes*	1	0.05	0.04
*Planctomycetes*	0	0.00	0.06
*Deinococcus-Thermus*	0	0.00	0.05
*Chlamydiae/Verrucomicrobia*	0	0.00	0.02
*Synergistetes*	0	0.00	0.09
*Thermotogae*	0	0.00	0.05
*Nitrospirae*	0	0.00	0.01
Phage/Plasmid/Virus	6	0.32	0.49
*Eukaryota*	17	0.89	1.45

We further compared the genome of *H. bizzozeronii *CIII-1 with the genomes of 10 human *H. pylori *strains to identify proteins that are unique to *H. bizzozeronii *as well as proteins found in *H. pylori *but lacking in *H. bizzozeronii*. The *H. pylori *core genome and *H. bizzozeronii *CIII-1 have 1,034 groups of orthologues in common, to which 1,052 CDSs from CIII-1 belong. Moreover, 412 groups are found in *H. bizzozeronii *CIII-1 and in at least two *H. pylori *strains, and 192 groups are common in CIII-1 and in at least one *H. pylori *strain. The *H. pylori *core genome contains 151 groups of unique orthologues while a total of 562 CDSs are unique to *H. bizzozeronii *CIII-1. Among the 562 unique *H. bizzozeronii *CDSs, a putative function was predicted for only 147 (26%). Each of these 147 CDSs was searched against the NCBI nr database using BLASTp to identify homologies with other *H. pylori *strains. A total of 59 out of the 147 CDSs showed a homology to proteins of at least one *H. pylori *strain, restricting the number of unique *H. bizzozeronii *CDSs with a predicted function to 88. Excluding the proteins associated either with bacteriophage or with plasmid replication, the functions of the majority of the unique *H. bizzozeronii *genes are linked to chemotaxis and metabolism (Figure 1).

### General features involved in the colonisation of the stomach: acid acclimation, mobility and chemotaxis

As described for the *H. pylori *genome [18], the *H. bizzozeronii *CIII-1 genome contains a complete urease gene cluster (*ure*ABIEFGH; HBZC1_10430 to HBZC1_10490) that is essential for acid acclimation and pH homeostasis, crucial mechanisms for the gastric colonisation. The urease gene cluster showed the same syntheny as in *H. pylori*. Unlike the other animal gastric *Helicobacter *species, *H. felis *and *H. mustelae *[13,16], *H. bizzozeronii *CIII-1 lacks the additional urease genes (*ure*AB2). Other essential mechanisms for the colonisation of the gastric mucosa participate in pH homeostasis [18]: *H. bizzozeronii *CIII-1 contains the periplasmic α-carbonic anhydrase orthologue HBZC1_14670, which contributes to the urease-dependent response to acidity in *H. pylori *[19], but lacks an orthologue for the β-carbonic anhydrase. In addition, *H. bizzozeronii *CIII-1 carries both the asparagine and glutamine deamidase-transport systems of AnsB (HBZC1_05200 and HBZC1_12160) plus DcuA (HBZC1_00140) and γGT (HBZC1_08080) plus GltS (HBZC1_14360) that contribute to periplasmic ammonia production in *H. pylori *and may have a possible role in the resistance to weakly acidic conditions and in the pathogenesis of gastritis [20]. A unique feature of *H. bizzozeronii *CIII-1 that might also contribute to acid acclimation is the presence of two copies of a putative allophanate hydrolase (HBZC1_04550 and HBZC1_09470). This enzyme catalyses the second reaction of the two-step degradation of urea to ammonia and CO_2 _and is usually found adjacent to a biotin-containing urea carboxylase gene (HBZC1_04560 and HBZC1_09490, respectively), which functions as an allophanate synthase from urea [21]. Orthologues of allophanate hydrolase have been found to be widely distributed among several subdomains of Bacteria, including *Campylobacter *species and *H. felis*, but their role in urea degradation has been demonstrated only in the environmental α-proteobacteria species *Oleomonas sagaranensis *[21]. In *H. bizzozeronii *the orthologue could contribute to the cytoplasmic degradation of urea in combination with urease. However, other functions, such as the degradation of aromatic compounds [22], could not be excluded.

Motility is fundamental for colonisation of the mucosa by gastric *Helicobacter *species [23], and, similarly to other flagellated bacteria, *H. bizzozeronii *CIII-1 harbours a large group of genes distributed widely in the genome, encoded by the *fla*, *flg*, *flh *and *fli *gene families, which are involved in the regulation, secretion and assembly of flagella [24].

Chemotactic pathways that are governed by two-component regulatory systems, histidine kinase proteins and regulatory proteins, modulate the direction and speed of motility using responses of specific signals detected by receptors formed by methyl-accepting chemotaxis proteins (MCPs). The repertoire of the chemotactic signal pathways of a microorganism is correlated to the environments it can transfer, the hosts it colonises and the diseases it can cause [25]. Despite the similar number of two-component systems, 4 histidine kinases and 10 regulatory proteins [26], *H. bizzozeronii *harbours five times more MCPs than *H. pylori*. Twenty MCPs, one containing a PAS domain, were detected in the *H. bizzozeronii *CIII-1 genome compared to the four predicted in *H. pylori *26695 (Figure 1; see additional file 1: Table S1) [26]. The abundance of predicted MCPs, similarly observed in *H. felis *[16], indicates an elaborate sensing capability of *H. bizzozeronii *that allows the bacterium to survive in different ecological niches and probably supports its capability to transfer between different hosts.

### The central intermediary metabolism

Understanding the metabolic properties of any pathogen is crucial to gain a full picture of the pathogenicity of related diseases. For example, *C. jejuni *is a more versatile and metabolically active pathogen than the more specialised *H. pylori*. The metabolic versatility of *C. jejuni *enables the bacterium to survive in different environments and to colonise a variety of animal species, while the high specialisation of *H. pylori *is probably the result of a process of adaptation to the human stomach [27]. Although *H. pylori *and *H. bizzozeronii *colonise similar niches, an important difference between these two species is the capability of *H. bizzozeronii *to move from a dog to a human host [2]. The central metabolism of *H. bizzozeronii*, which can be inferred from the genome of the human strain CIII-1, appears to be more flexible than that of *H. pylori *and could explain the zoonotic nature of this species.

As observed for *H. pylori *[28,29], even though the glycolysis pathway is incomplete because of the lack of phosphofructokinase, *H. bizzozeronii *CIII-1 may use glucose as a source of energy through the pentose phosphate reactions and the Entner-Doudoroff pathway. However, in contrast to what was observed in *H. pylori*, *H. bizzozeronii *CIII-1 possess three genes organised in a single operon that are required for glycerol metabolism: glycerol-3-phosphate dehydrogenase (*glpD*, HBZC1_03150), glycerol kinase (*glpK*, HBZC1_03160 and HBZC1_03170) and a glycerol uptake facilitator protein (*glpP*, HBZC1_03180). Glycerol-3-phosphate is a central intermediate in glycolysis and in phospholipid metabolism [30]. The enzyme glycerol-3-phosphate dehydrogenase converts glycerol-3-phosphate to glyceraldehyde-3-phosphate, which can enter the glycolysis pathway, conferring significant metabolic advantages on this species.

**Figure 2:**
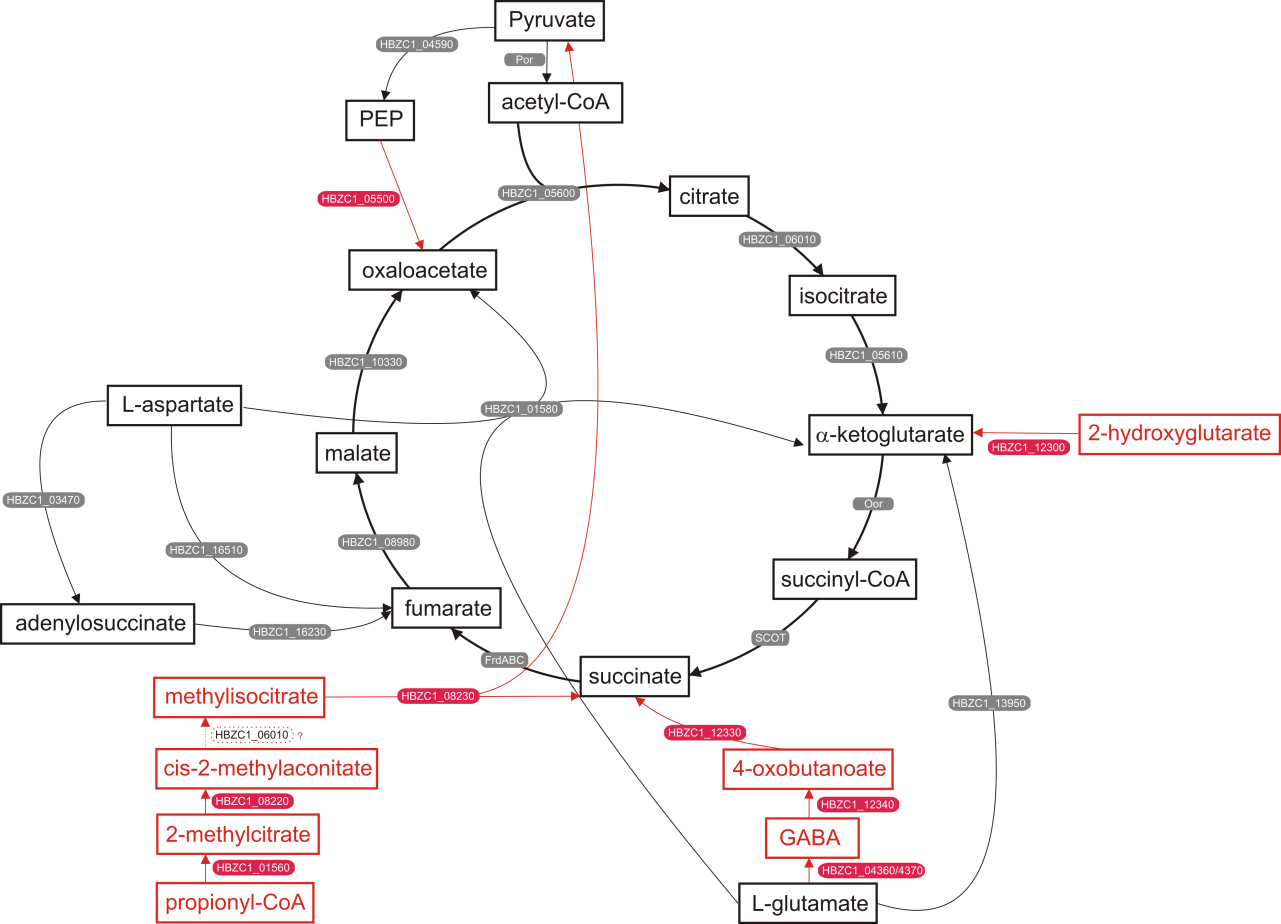
**Schematic representation of the central metabolism (predicted citric acid cycle and anaplerotic reactions) of *Helicobacter bizzozeronii *CIII-1**. In red the reactions predicted to be present in *H. bizzozeronii *CIII-1 but not in *H. pylori*. In black the reactions predicted to be in common in both *Helicobacter *species. Reaction for which no predictable enzymes were found in the genome is indicated by dotted line. The *H. bizzozeronii *gene numbers are indicated in boxes.

The absence of obvious homologues of succinyl-CoA synthetase indicates the presence of the alternative citric acid cycle (CAC), which has been described in *H. pylori *[31] (Figure 2; see additional file 1: Table S2). However, in contrast to *H. pylori *[27], the *H. bizzozeronii *CIII-1 genome contains several genes involved in the anaplerotic replenishment of oxaloacetate, succinate and α-ketoglutarate (Figure 2; see additional file 1: Table S2). Like *C. jejuni *[27], the *H. bizzozeronii *CIII-1 genome includes a homologue of phosphoenol pyruvate (PEP) carboxylase (HBZC1_05500) that may function in oxaloacetate synthesis. In addition, the genome harbours genes encoding enzymes potentially involved in the 2-methylcitric acid cycle (HBZC1_01560; HBZC1_08220; HBZC1_08230; HBZC1_06010) that produces succinate and pyruvate from the breakdown of propionate [32]. Moreover, a group of three genes, 4-aminobutyrate transaminase (HBZC1_12340), NADP+-Succinate-semialdehyde dehydrogenase (HBZC1_12330) and an amino acid permease (HBZC1_12320), share homology with the *gabDTP *operon of *E. coli *but do not have orthologues in any of the *H. pylori *sequenced genomes. These genes are potentially involved in the production of succinic acid from the degradation of polyamines or γ-aminobutyric acid (GABA) [33]. The synthesis of GABA from L-glutamate might occur in *H. bizzozeronii *trough the action of a glutamate decarboxylase (HBZC1_04360/HBZC1_04370) which does not have orthologue in *H. pylori*. Downstream of the putative *H. bizzozeronii gabDTP *operon, there are two gene homologues to the *E. coli *carbon starvation-induced protein (*csiD*, HBZC1_12310) and L-2-hydroxygluturate oxidase (*lhgO*, HBZC1_12300). In *E. coli*, *lhgO*, located immediately downstream of *csiD *and upstream of the *gabDTP *operon, plays a role the recovering α-ketoglutarate, an intermediate in CAC, reduced by other enzymes [34]. In *E. coli*, all of these genes are regulated by the CsiR repression and σ^S ^induction acting on *csiD_p _*during carbon starvation and the stationary phase [35]. *H. bizzozeronii *CIII-1 lacks both of these transcriptional regulators but it contains a homologue of carbon-starvation regulator (CsrA, HBZC1_09070) that controls the *H. pylori *response to environmental stress [36] and could play a similar role in *H. bizzozeronii*.

Finally, *H. bizzozeronii *CIII-1 harbours a cluster of 4 conserved genes (HBZC1_00410 to HBZC1_00460), annotated as acetophenone carboxylase subunits 1 to 4, which show a low homology with microbial hydantoinases. This group of genes is located near to the SCOT (*scoAB*, HBZC1_00390 and HBZC1_00400) and acetoacetyl-CoA thiolase (*fadB*, HBZC1_00380) genes. A similar gene cluster, encoding a set of enzymes capable of metabolizing acetone to acetyl-CoA, has also been described in *H. pylori *(*scoAB*, *fadB *and *acxABC*) [37]. However, the homology between the *H. pylori acxABC *and the *H. bizzozeronii *acetophenone carboxylase gene cluster is very low (<30%), indicating that the *H. bizzozeronii *genes could be involved in an alternative pathway.

### Physiology of microaerobic growth

**Figure 3:**
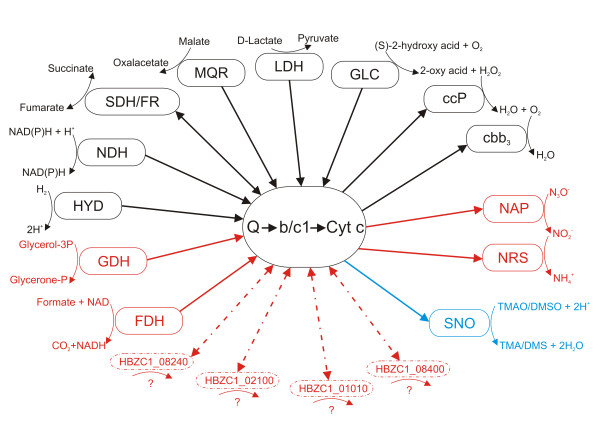
**Schematic representation of the respiratory pathway in *Helicobacter bizzozeronii *CIII-1**. In red the reactions predicted to be present in *H. bizzozeronii *CIII-1 but not in *H. pylori*. In black the reactions predicted to be in common in both *Helicobacter *species. In blue TMAO/DMSO reduction which could be predicted also in *H. pylori *but a complete set of enzyme is not present. The arrows indicate the direction of the electrons to or from the membrane-bound electron transport chain. Dashed lines indicate that the direction of the electrons is unknown. Enzymes catalyzing key reactions are indicated in boxes. FDH, formate dehydrogenase; HYD, hydrogenase; NRS, nitrite reductase system; SNO, S- and N- oxidases; GDH, glycerol-3-phosphate dehydrogenase; NDH, NAD(P)H dehydrogenase; SDH/FR, succinate dehydrogenase/fumarate reductase; MQR, malate-quinone-reductase; LDH, lactate dehydrogenase; GLC, glycol oxidase; NAP, periplasmic nitrate reductase; Q, quinone; Cyt b/c1, quinone cytochrome oxidoreductase; Cyt c, cytochrome c; cbb3, cytochrome c oxidase; ccP, cytochrome c peroxidase.

The complexity of the electron transport chain is another important element in the ability of bacteria to grow under a variety of environmental conditions, determining the metabolic flexibility of the bacterium based on the variety of electron donors and acceptors that can be used to support growth [38]. The respiratory chain in *H. bizzozeronii *appears to be highly branched and more complex than described for *H. pylori *(Figure 3; see additional file 1: Table S2).

In addition to hydrogenase HydABCDE (from HBZC1_10200 to HBZC1_10150) and other dehydrogenases in common with *H. pylori *(Figure 3; see additional file 1: Table S2) [39], the *H. bizzozeronii *CIII-1 genome contains a putative formate dehydrogenase enzyme complex, organised in a operon (from HBZC1_13510 to HBZC1_13550), which could enable the bacteria to use formate as an electron donor, as observed in other ε-proteobacteria [38]. In addition, glycerol-3-phosphate dehydrogenase (HBZC1_03150) provides *H. bizzozeronii *CIII-1 an extra possibility for substrate-derived electrons to be donated to the membrane-bound electron transport chain.

*H. bizzozeronii *requires oxygen for growth and is unable to survive under anaerobic conditions [9]. As previously observed in *H. pylori*, *H. bizzozeronii *CIII-1 possesses only one terminal oxidase, *ccoNOQP *(from HBZC1_08510 to HBZC1_08540), which encodes a cytochrome *cb*-type enzyme, the major factor in aerobic respiratory metabolism [38]. In addition to using an oxygen-dependent electron transport, *H. bizzozeronii *CIII-1 could use as electron acceptors fumarate by a fumarate reductase system (FrdABC, from HBZC1_01900 to HBZC1_01920), as described in *H. pylori *[39]. However *H. bizzozeronii *could also utilize nitrate and nitrite as electron acceptors. This characteristic had been already shown in *C. jejuni *but not in *H. pylori *[38]. Nitrate could be reduced by a periplasmic nitrate reductase system (NapAGH, from HBZC1_08800 to HBZC1_08830; NapB, HBZC1_04310; NapFD; HBZC1_03530-HBZC1_03540), which differs from the *C. jejuni *system for the absence of NapL homologues and by the fact that it is not organised in a single operon (Figure 1) [38]. Moreover, although a clear *C. jejuni *nitrite reductase orthologue is absent, the genome of *H. bizzozeronii *CIII-1 contains a cluster of two genes encoding a putative periplasmic nitrite reductase (HBZC1_10540) and a cytochrome-c nitrite reductase subunit c552 domain containing protein (annotated as hydroxylamine oxidoreductase; HBZC1_10550), suggesting that nitrate could be used in anaerobic respiration by this species. In addition to using nitrate and nitrite, *H. bizzozeronii *could also use S- or N-oxides as electron acceptors, as observed in other ε-proteobacteria [27]. In fact, trimethylamine-n-oxide (TMAO) and dimethyl sulfoxide (DMSO) could be reduced by a putative periplasmic system, a homologue of Cj0264c/Cj0265c (HBZC1_10800/HBZC1_10810) [38]. In *H. pylori *a putative trimethylamine-N-oxide reductase is present (HP0407 in *H. pylori *26695; 41% amino acid identity with HBZC1_10810 over 93% of sequence length). Although a homologue of the monoheme *c *type cytochrome (HBZC1_10800/Cj0265c) is absent, we cannot exclude that *H. pylori *may also be able to use TMAO/DMSO in respiration through a different pathway.

Other genes possibly involved in the electron transport chain that are present in the *H. bizzozeronii *CIII-1 genome without clear homologues in *H. pylori *are: two copies of an NAD(P)H-dependant oxidoreductase (HBZC1_02100 and HBZC1_08240), a pyridine nucleotide-disulphide oxidoreductase (HBZC1_01010) and an aldo/keto reductase (HBZC1_08400). Although it is not possible to predict the functions of these genes, their presence increases the complexity of the electron transport chain of *H. bizzozeronii *and clearly differentiates this species from *H. pylori*.

Like *H. pylori *[39], because of its microaerophilic requirements, *H. bizzozeronii *expresses several defence mechanisms against oxidative stress. These include an iron-cofactored superoxide dismutase SodB (HBZC1_11160/11150), catalase enzyme KatA (HBZC1_12240), Cytocrome-c peroxidase (HBZC1_05800) and alkyl hydroperoxide reductase (HBZC1_01800). Moreover, because metal ions like iron and nickel favour the creation of reactive oxygen species (ROS), the detoxification of ROS needs to be coupled tightly to the control of intracellular metal concentrations, ensuring metal ion homeostasis by the regulation of metal uptake, export and storage [40]. *H. bizzozeronii *contains all of the genes involved in nickel and iron uptake, storage and metabolism that have been described in other *Helicobacter *species [13,40].

### Features involved in the interaction with the host: surface structures and secreted proteins

*H. bizzozeronii *is able to persistently colonise the stomach of both dogs [2] and humans [6-8]. Therefore, the bacteria need to constantly interact with the hosts directly, using fixed bacterial surface molecules that mediate adherence, and indirectly, by the secretion of soluble proteins that act on respective host receptors.

Outer membrane proteins (OMPs) play important roles in the interaction with the host by mediating the adhesion to the mucosa and, therefore, the colonisation of the stomach and modulating the host immune response. Moreover, OMPs are essential for the general metabolism of the bacteria, preserving the selective permeability of the outer membrane to different substrates [41]. The *H. bizzozeronii *CIII-1 genome encodes a total of 51 putative OMPs (see additional file 1: Table S3) that are phylogenetically related to the five OMP families described in *H. pylori *[42]. Twenty of the *H. bizzozeronii *OMPs are similar to the Family 1 OMPs of *H. pylori *that include both Hop and Hor proteins. In contrast to the common finding that *H. pylori *attaches to the membrane of epithelial cells using different adhesive structures [42], studies on non-pylori *Helicobacter *species in mice and Mongolian gerbils [11,43] and in dogs [44] showed no evidence of a tight attachment to gastric epithelial cells by *H. bizzozeronii*. In fact, although 8 out a total of 20 *H. bizzozeronii *Family 1 OMPs belong to the Hop category, none of them could be considered as orthologues of the BabA, BabB or SabA adhesins, which play a major role in *H. pylori *adhesion to the gastric mucosa [45]. However, *H. bizzozeronii *was found in the intracellular canaliculi and in the cytoplasm of the parietal cells of canine gastric mucosa [44]. Although it is not clear how this *Helicobacter *sp. penetrates into the cytoplasm of parietal cells, a possible role of the *H. bizzozeronii *Hop proteins as adhesins with a different receptor specificity from that described in *H. pylori*, should be considered. Excluding the *hofA *homolog (HBZC1_6200), the other eight *H. bizzozeronii hof *genes are organised in a single operon (*hof*HHBFEGCD; from HBZC1_06760 to HBZC1_06830). This region is characterised by an anomalously low GC content and was identified as potential laterally acquired DNA by the Alien Hunter program [46]. Hof family was identified from the *H. pylori *proteome based on a heat-stable 50 KDa OMP [41], but its role is still unknown. Three of the *H. bizzozeronii *OMPs belong to Hom Family 3, and a total of 5 putative efflux pump OMPs (Family 5) were identified. Moreover, 5 iron-regulated OMPs (Family 4) were detected in the *H. bizzozeronii *genome. Finally, the remaining 10 *H. bizzozeronii *OMPs are currently unclassified, including the putative VacA-paralog (HBZC1_11860/11850), a peptidoglycan-associated lipoprotein (HBZC1_14500), the membrane-associated lipoprotein Lpp20 (HBZC1_01250) and a putative protease (HBZC1_01500).

The communication between a microbial pathogen and its host occurs also due to the recognition of cell surface glycomolecules, including lipopolysaccharides (LPS), capsular polysaccharides and N- and O-glycoproteins, the structure of which depends of the presence and functional transcription of the genes encoding glycosyltransferases (GTs) [47]. A total of 24 GTs were annotated in the genome of *H. bizzozeronii *CIII-1 (see additional file 1: Table S4), 22 of which belong to 10 distinct GT-families according to the sequence-based classification of GTs available in the CAZy database [48]. In addition, the genome of *H. bizzozeronii *CIII-1 harbours 20 orthologues of genes implicated in the biosynthesis of glycan structures in other bacterial species (see additional file 1: Table S4), including a complete flagellin O-glycosylation pathway that is essential for flagella assembly and motility [49]. The LPS of *H. pylori*, which displays Lewis blood-group (Le) antigens, is a primary host-interacting structure, and several lines of evidence indicate that it is involved in host-adaptation and virulence [50]. Distinct from *H. pylori*, *H. bizzozeronii *produces a predominantly low-molecular-weight LPS and does not apparently exhibit a molecular mimicry of Lewis and other blood group antigens [51]. However, anti-*H. pylori *LPS core antibodies reacted with the *H. bizzozeronii *LPS, indicating a shared epitope between these two species [51]. Several genes are involved in the biosynthesis of LPS in *H. bizzozeronii *and are dispersed across the genome as they are described in *H. pylori *[26]. In addition to the conserved genes of the LPS biosynthesis pathway (see additional file 1: Table S4), the *H. bizzozeronii *CIII-1 genome includes a putative α1,3-fucosyltransferase (GT10; HBZC1_11830), a tandem of two putative galactosyltransferases (GT25; HBZC1_4780/4770/4760 and HBZC1_4800/4790) that are fragmented due to the presence of homopolymeric runs, a putative α1,3-sialyltransferase (GT42; HBZC1_02560) and other putative glycosyltransferases that belong to GT-family 2 or 4 or are unclassified. Structural analysis of the *H. bizzozeronii *LPS have shown the presence of fucose [51] and sialyl-lactoseamine (M. Rossi, unpublished data), confirming the role of GT10, GT25 and GT42 in the modification of the oligosaccharide region of LPS. However, several other GTs cannot be confidently included in a specific biosynthesis pathway. Among these genes, a cluster of 6 GTs (from HBZC1_07440 to HBZC1_07490) and a putative polysaccharide deacetylase (HBC1_07500) are particularly interesting (Figure 1). Orthologues of these genes are not found in *H. pylori*. Although some of these genes have homologues in the *H. felis *and *H. suis *genomes [15,16], the number and synteny of the genes appear to be specific characteristics of *H. bizzozeronii*.

The extracellular proteins of *H. pylori *are known to mediate pathogen-host interaction during infection [52]. To export proteins *H. bizzozeronii*, as a gram-negative bacterium, utilises the Sec or the twin-arginine secretion (Tat) systems to transport proteins into the periplasmic space and uses different approaches to move the proteins through the outer membrane [53]. In *H. bizzozeronii *CIII-1, the Sec system, which lacks a homologue to SecB, is dispersed through the genome, as it is in other *Helicobacter *spp. [13,53] and the Tat system is composed of three genes that are homologous to the TatABC traslocation proteins TatA (HBZC1_10870) and YatBC (HBZC1_10370-10380) and a putative deoxyribonuclease that is orthologous to *H. pylori tatD *(HBZC1_14750). Both of the systems recognise an N-terminal signal peptide of the protein that is required for translocation [53]. More than 200 proteins were predicted to have a Sec signal peptide using the SignalP software [54] (data not shown). However, only 50 proteins, 22 of which harbour a signal peptide, appear to be located in the periplasmic space or outside of the outer membrane, as predicted using the PSORTb v3 tool [55] (Table 3). Nevertheless, the subcellular locations of more than 600 *H. bizzozeronii *CIII-1 proteins were not identified. Several proteins that share a clear homology with excreted proteins in *H. pylori *were predicted to be located in the cytoplasm, indicating that the repertoire of secreted proteins in *H. bizzozeronii *could be considerably different from what predicted using PSORTb.

**Table 3:** List of putative *Helicobacter bizzozeronii *secreted proteins predicted by PSORTbv3

CDS Locus_Tag	Predicted function	Localization	Sec SP^1^	Tat SP^2^	SpII^3^	*H. pylori *orthologue CDS Locus_tag
HBZC1_00340	Oligopeptide ABC transporter, periplasmic oligopeptide-binding protein OppA	Periplasmic	+	-	-	HP1252
HBZC1_00890	Hypothetical protein (sel 1 domain repeat containing protein)	Extracellular	-	-	-	(-)^4^
HBZC1_00900	Hypothetical protein (sel 1 domain repeat containing protein)	Extracellular	+	-	-	(-)
HBZC1_00910	Hypothetical protein (sel 1 domain repeat containing protein)	Extracellular	+	-	-	(-)
HBZC1_01330	Flagellar hook protein FlgE	Extracellular	-	-	-	HP0870
HBZC1_01540	Flagellar hook-associated protein FlgK	Extracellular	-	-	-	HP1119
HBZC1_01610	HtrA protease/chaperone protein/Serine protease	Periplasmic	+	-	-	HP1019
HBZC1_02310	Membrane-anchored cell surface protein	Extracellular	-	-	-	(-)
HBZC1_02430	Hypothetical protein (sel 1 domain repeat containing protein)	Extracellular	+	-	-	(-)
HBZC1_03080	Flagellar basal-body rod protein FlgC	Periplasmic	-	-	-	HP1558
HBZC1_03090	Flagellar basal-body rod protein FlgB	Periplasmic	-	-	-	HP1559
HBZC1_04310	Nitrate reductase cytochrome c550-type subunit	Periplasmic	+	-	-	(-)
HBZC1_04340	Flagellin	Extracellular	-	-	-	HP0115
HBZC1_05160	Soluble lytic murein transglycosylase	Periplasmic	+	-	-	HP0649
HBZC1_05800	Cytochrome c551 peroxidase	Periplasmic	+	-	-	HP1461
HBZC1_05960	Putative amino-acid transporter periplasmic solute-binding protein	Periplasmic	+	-	+	HP1172
HBZC1_06690	Sel1 domain-containing protein repeat-containing protein	Extracellular	-	-	-	(-)
HBZC1_07110	Flagellar hook-associated protein FlgL	Extracellular	-	-	-	HP0295
HBZC1_07220	Hypothetical protein	Extracellular	+	-	+	(-)
HBZC1_07230	Hypothetical protein	Extracellular	-	-	+	(-)
HBZC1_07880	Hypothetical protein	Extracellular	-	-	-	(-)
HBZC1_08080	Gamma-glutamyltranspeptidase	Periplasmic	+	-	-	HP1118
HBZC1_08620	Flagellar hook-length control protein FliK	Periplasmic	-	-	-	(-)
HBZC1_08630	Flagellar basal-body rod modification protein FlgD	Periplasmic	-	-	-	HP0907
HBZC1_08640	Flagellar hook protein FlgE	Extracellular	-	-	-	HP0908
HBZC1_08800	Periplasmic nitrate reductase	Periplasmic	-	+	-	(-)
HBZC1_08840	Flagellar P-ring protein FlgI	Periplasmic	+	-	-	HP0246
HBZC1_09700	Hypothetical protein (sel 1 domain repeat containing protein)	Extracellular	+	-	+	HPSH_03725
HBZC1_10550	Cytochrome c nitrite reductase subunit c552	Periplasmic	+	-	-	(-)
HBZC1_10810	trimethylamine-N-oxide reductase	Periplasmic	+	-	-	HP0407
HBZC1_10890	mJ0042 family finger	Extracellular	-	-	-	(-)
HBZC1_11140	Hypothetical protein (sel 1 domain repeat containing protein)	Extracellular	+	-	-	(-)
HBZC1_11160	Superoxide dismutase	Periplasmic	-	-	-	HP0389
HBZC1_11330	YgjD/Kae1/Qri7 family protein	Extracellular	-	-	-	HP1584
HBZC1_11450	Flagellar hook-associated protein FliD	Extracellular	-	-	-	HP0752
HBZC1_12200	Hypothetical protein	Extracellular	-	-	-	(-)
HBZC1_12220	Hypothetical protein	Periplasmic	+	-	-	(-)
HBZC1_12240	Catalase	Periplasmic	-	-	-	HP0875
HBZC1_12430	Nucleoside diphosphate kinase	Extracellular	-	-	-	HP0198
HBZC1_13860	Cytochrome C553 (soluble cytochrome f)	Periplasmic	+	-	-	jhp1148
HBZC1_13880	Flagellin	Extracellular	-	-	-	HP0601
HBZC1_14010	Outer membrane protein	Extracellular	-	-	-	(-)
HBZC1_14040	Flagellar basal-body rod protein FlgG	Extracellular	-	-	-	HP1092
HBZC1_14510	TolB protein	Periplasmic	+	-	-	HP1126
HBZC1_14760	Membrane-bound lytic murein transglycosylase D	Periplasmic	+	-	-	HP1572
HBZC1_15010	Arginine decarboxylase	Periplasmic	-	-	-	HP0422
HBZC1_16410	Hypothetical protein (sel 1 domain repeat containing protein)	Extracellular	-	-	-	(-)
HBZC1_16420	Hypothetical protein (sel 1 domain repeat containing protein)	Extracellular	-	-	-	(-)
HBZC1_17210	Hypothetical protein	Periplasmic	-	-	-	HPG27_960
HBZC1_17260	Endonuclease I	Extracellular	-	-	-	(-)

^1^The Sec specific signal peptide predicted using SignalP-HMM (http://www.cbs.dtu.dk/services/SignalP/); probability score cut off 0,500

^2^The Tat specific signal peptide predicted using TatP 1.0 Server (http://www.cbs.dtu.dk/services/TatP/)

^3^The signal peptide SpII of lipoproteins was predicted using LipoP 1.0 Server (http://www.cbs.dtu.dk/services/LipoP/)

^4^No orthologues in *H. pylori*; BLASTP against nr database: two proteins with amino acid identity greater than 40% over 80% sequence length were considered homologues

It has been shown in *H. pylori *that some proteins could be exported from the cytoplasm to the extracellular milieu or directly to the cytoplasm of the host through Sec-independent secretion systems [53]. *H. bizzozeronii *CIII-1 genome lacks orthologues of CagPAI that encode a type IV secretion system responsible in *H. pylori *of the translocation of the effector protein CagA into the cytosol of the host [23]. In addition to containing the secretion system that exports the flagella subunit across the membrane, *H. bizzozeronii *CIII-1 harbours a type IV secretion systems derived from genes orthologues of *com*B2, 3, 4, 5, 6, 7, 8, 9 and 10 that are organised in different operons dispersed throughout the genome (Figure 1; see additional file 1: Table S5). In *H. pylori*, the *comB *components are required for uptake DNA by natural transformation [56], and its orthologues likely encode same function in *H. bizzozeronii*.

*H. bizzozeronii *is able to induce similar injuries in the human gastric mucosa as is *H. pylori*, indicating that both of the species express similar virulence factors. Although VacA and CagA homologues are missing, other virulence-associated genes described in *H. pylori *are present in the *H. bizzozeronii *CIII-1 genome and typically share a high degree of similarity. These virulence factors could contribute to the disease outcome and include the following: the immunomodulator NapA (HBC1_08880); the peptidyl propyl-cis,trans-isomerase (HBZC1_06110), involved in the TLR4-dependent NF-κB and in AP1 activation of macrophages [57]; the tumour necrosis factor alpha-inducing protein (Tipα, HBZC1_02220), described in *H. pylori *as a carcinogenic factor [58]; and the secreted serine protease (HtrA, HBZC1_01610), which cleaves the ectodomain of the cell-adhesion protein E-cadherin providing an access to the intercellular space for *H. pylori *[59] (Figure 1). A putative virulence factor in *H. bizzozeronii *CIII-1 that does not have homologues in any *H. pylori *is HBZC1_15820, encoding a polysaccharide lyase belonging to Family 8 (Figure 1) [48]. A similar gene was found in the genome of *H. felis *[16], but a homologue is absent in all of the other sequenced ε-proteobacteria. Polysaccharide lyases are eliminases that cleave acidic polysaccharides, such as glycosaminoglycans, at specific glycosidic linkages [60]. Family 8 includes hyaluronate, chondroitin AC/ABC and xanthan lyases that have been isolated from several bacterial species, both non-pathogenic and pathogenic, including *Bacillus *spp., *Penibacillus *spp., *Proteus *spp., *Clostridium *spp., *Staphylococcus *spp. and *Streptococcus *spp. [48]. In certain pathogenic species the polysaccharide lyases represent a virulence factor that is important, for example in *Staphylococcus aureus*, in the early stages of infections [61]. The role of this putative polysaccharide lyase in both *H. bizzozeronii *and *H. felis *is completely unknown and requires further study.

### Genome plasticity

*H. bizzozeronii *is well adapted to colonise the stomach of dogs [44] and has probably coevolved with its natural host, as has been demonstrated for other gastric *Helicobacter *species [1,14]. However, *H. bizzozeronii *is not strictly restricted to the canine gastric mucosa but is able to colonise different hosts [2]. Therefore, when transferring from dogs to humans, *H. bizzozeronii *necessarily undergoes intensive changes to adapt to a new host. As is the case for *H. pylori *[1], the ability of *H. bizzozeronii *for ongoing host-adaptation is related to its high level of genome plasticity.

The *H. bizzozeronii *CIII-1 genome lacks multiple genes in the methyl-directed mismatch repair system (MMR), similar to other ε-proteobacteria [13,62]. A consequence of such a defect in the MMR is the formation and extension of multiple hypervariable simple sequence repeats (SSRs), potentially responsible for the phase variation in *H. bizzozeronii*. SSRs are subject to slipped-strand mutations, therefore inducing frameshift of the gene if the repeats are located in a protein-coding region or alteration of the gene expression when the repeats affect the promoter [63]. It has been shown that the physiological role of SSRs may not be limited to phase variation, but can include reorganisation of the chromosome, influence on protein structure and function, and possible antisense regulation [16,64,65]. A total of 80 SSRs, 71 of which are homopolymeric runs of ≥9 units of a single nucleotide, were detected dispersed across the *H bizzozeronii *CIII-1 genome, with 64 (the majority) located inside the coding regions. The frames of 54 genes are affected by the slipped-strand mispairing associated with SSRs. After the exclusion of essential genes which are unlikely subjected to phase variation by slipped-strand mispairing (i.e., tRNA-guanine transglycosylase, cell division protein FtsK, DNA polymerase and spermidine synthase) we were able to identify a total of 43 potentially phase-variable genes (Table 4). Similar number of contingency genes was also described in *H. pylori *[66], and, as observed in *C. jejuni *and *H. pylori*, the dominant function of the contingency genes in *H. bizzozeronii *CIII-1 is the modification at either the protein or carbohydrate level of the surface architecture, suggesting that these structures play an important role in the adaptation to different hosts [67,68].

**Table 4:** Putative phase variable genes of *Helicobacter bizzozeronii *CIII-1

	Repeat unit	Length(bp)	Region	Localization^1^	CDS Locus_Tag^2^	Predicted function
Glycan biosynthesis						
	C	20	1,090,089..1,090,108	5'	HBZC1_11830	Alpfa(1,3)-Fucosyltransferase (glycosyltransferase Family GT10)
	C	19	448,597..448,615	M	HBZC1_04780/HBZC1_04770/HBZC1_04760	Putative Beta-Galactosyltransferase (glycosyltransferase Family GT25)
	C	19	449,444..449,462	5'	HBZC1_04800/HBZC1_04790	Putative Beta-Galactosyltransferase (glycosyltransferase Family GT25)
	C	14	237,793..237,806	5'	HBZC1_02530/HBZC1_02540	UDP-N-acetylglucosamine 2-epimerase
	G	13	339,856..339,872	M	HBZC1_03690/HBZC1_03680	Glycosyltransferase Family GT4
	CT	13	1,310,060..1,310,085	M	HBZC1_14140	Glycosyltransferase Family GT4
	CACACAA	13	706,715..706,800	M	HBZC1_07440	Glycosyltransferase Family GT4
Cell-surface-associated proteins						
	G	15	478,890..478,904	5'	HBZC1_05060	Hypothetical protein (putative HcpA)
	G	10	732,027..732,038	M	HBZC1_07730/HBZC1_07740	CBS domains containing protein (homologue of HP1490 - TolC efflux pump)
	G	15	1,677,616..1,677,630	5'	HBZC1_18160	Putative ATP/GTP binding protein
	G	9	58,965..58,973	5'	HBZC1_00570	Massive surface protein (MspC)
	G	9	640,616..640,624	M	HBZC1_06770	Outer membrane protein
	C	9	809,713..809,721	M	HBZC1_08590/HBZC1_08580	Outer membrane protein (omp30)
	A	9	1,401,250..1,401,258	5'	HBZC1_15140/HBZC1_15150	Outer membrane protein
	C	9	666,243..666,252	M	HBZC1_06970	Putative paralog of HpaA
	C	9	880,014..880,022	5'	HBZC1_09290	Hypothetical protein
	T	9	167,433..167,441	M	HBZC1_01860	Cardiolipin synthetase (phosholipase D)
	C	9	1,364,186..1,364,194	5'	HBZC1_14770	Rare lipoprotein A precursor
	C	9	611,706..611,714	5'	HBZC1_06460	Methyl-accepting chemotaxis protein
	G	9	831,049..831,057	5'	HBZC1_08750/HBZC1_08760	Flagellar assembly protein FliH
	A	9	813,507..813,515	5'	HBZC1_08620	Flagellar hook-length control protein FliK
	C	9	1,380,513..1,380,521	M	HBZC1_14960	Hypothetical protein (probable membrane protein)
	G	9	1,581,243..1,581,251	5'	HBZC1_17050	Omp
	AG	8	516,821..516,838	M	HBZC1_05450/HBZC1_05460	Membrane protein containing sulfatase domain
	TC	6	1,716,985..1,716,996	M	HBZC1_18530	Membrane protein containing sulfatase domain
	CAGCA	14	1,532,941..1,533,010	5'	HBZC1_16470	HopZ
Restriction/Modification system						
	AC	20	823,472..823,513	3'	HBZC1_08670/HBZC1_08280	Type I restriction-modification system, DNA-methyltransferase subunit M and S
Stress associated proteins						
	C	9	204,956..204,964	M	HBZC1_02170	Cold-shock DEAD-box protein A
	G	10	1,343,484..1,343,493	M	HBZC1_14490	Putative periplasmic protein contains a protein prenylyltransferase domain
Other proteins						
	G	9	886,063..886,071	M	HBZC1_09350/HBZC1_09360	Hypothetical protein (homolog of putative heterodisulfide reductase *H. suis*)
	G	17	981,720..981,736	M	HBZC1_10620/HBZC1_10630	Hypothetical protein (Sel1 domain-containing protein)
	C	16	1,314,541..1,314,556	5'	HBZC1_14170/HBZC1_14180	Hypothetical protein
	G	9	177,594..177,602	M	HBZC1_01940	Hypothetical protein
	C	10	372,167..372,176	5'	HBZC1_04030	Hypothetical protein
	A	9	571,554..571,562	5'	HBZC1_06030	Hypothetical protein
	C	13	886,063..886,071	M	HBZC1_10080	Hypothetical protein
	C	17	986,120..986,136	M	HBZC1_10650	Hypothetical protein
	G	9	1,135,720..1,135,728	5'	HBZC1_12200	Cytidine monophosphate-N-acetylneuraminic acid contains Rieske domain
	C	14	1,235,708..1,235,721	5'	HBZC1_13300	Hypothetical protein
	C	9	1,380,536..1,380,544	M	HBZC1_14960	Hypothetical protein
	G	10	1,458,356..1,458,364	M	HBZC1_15990	Hypothetical protein
	G	9	1,571,406..1,571,414	M	HBZC1_16940	Hypothetical protein
	G	13	1,659,328..1,659,340	5'	HBZC1_17930	Hypothetical protein (contains LexA/Signal peptidase domain)

^1^localization of the SSR in the 5' terminal part (5'), 3' terminal part (3') or in the middle (M) of the corresponding CDS

^2^when modifying the repeat length allows to merge two or more adjacent CDSs, the respective locus tags are indicated in the table separated by a slash

The extreme genetic diversity observed among the *H. pylori *strains appears to also be generated by horizontal gene transfer (HGT) as a result of DNA recombination via natural transformation [1]. Foreign DNA acquired by HGT can be associated in bacteria with insertion sequence (IS) elements or tRNA genes and can be identified by an anomalous GC content or as a change of code usage [69]. The *H. bizzozeronii *CIII-1 genome harbours five IS elements, with two belonging to the IS200/IS605 family and three to the IS607 family according to the semi-automatic annotation system provided by the ISsaga tool [70]. Each IS element includes two genes, the *orfA *and *orfB *transposases. In addition, *H. bizzozeronii *CIII-1 contains 22 almost identical mini-IS elements of 232 bp with ~98% nucleotide identity uniformly distributed across the genome (Figure 1). The Mini-IS elements are vestigial remnants of full-length elements and have also been described in *H. pylori *[71,72]. Although it is not known if any of these elements have significant functional roles, the mini-ISs could be responsible for the alteration of the expression of some genes, and they may mediate HGT, thereby contributing to the genetic diversity among *H. bizzozeronii *strains.

Large inserts of laterally acquired DNA containing functionally related genes are often referred to as genomic islands (GIs) and are typically associated with an increased virulence [13]. The Island Viewer and Alien Hunter programs were able to identify a putative GI of ~70 kb (41 GC %) in the *H. bizzozeronii *CIII-1 genome from base 1,559,252 to 1,628,818 (Figure 1). The GI is flanked by two IS elements and contains 77 CDSs (see additional file 1: Table S6) of which a putative function was assigned to 24 (31%). The *H. bizzozeronii *GI does not include the type IV secretion system or known virulence factors, and some genes, such as conjugal transfer protein (HBZC1_17110), replication A protein (HBZC1_17000) and the plasmid partitioning protein ParA (HBZC1_16910), appear originally have been plasmid-related features. These results suggest that a transposition event may have been responsible for the integration of a plasmid in the chromosome as already observed in *C. jejuni *[73] and in *H. pylori *[74].

A putative self-replicating circular plasmid pHBZ1, 52 kb in length, was sequenced from *H. bizzozeronii *CIII-1 (FR871758). Among the 21 genes for which a putative function was predicted (see additional file 1: Table S7), the plasmid contains the following elements: two copies of an aldo-keto reductase (HBZC1_p0200 and HBZC1_p0210), a complete type I restriction modification system (from HBZC1_p0340 to HBZC1_p0380), a protein containing the HipA-domain (HBZC1_p0540; [75]) and two copies of an adenine-specific methyltransferase (HBZC1_p0660 and HBZC1_p0680). The distribution of this plasmid among other *H. bizzozeronii *strains and its role in the adaption to the host and pathogenesis are unknown and merit further study.

Finally, immediately downstream of the *H. bizzozeronii *GI, a putative prophage region of about 37 kb was identified using Prophage Finder program [76] and it harbours several elements with homology to *C. rectus *RM3267 phage genes (Figure 1). However, excluding those involved in the phage replication, the majority of the genes included in the prophage region did not have any predictable function.

## Conclusion

In this study, a detailed comparative genomic analysis between the human strain *H. bizzozeronii *CIII-1 and *H. pylori *showed important differences between these two human gastric pathogens, providing new insights into the comprehension of the biology of gastric *Helicobacter *species. Although *H. bizzozeronii *possesses all genes necessary for its specialized life in the stomach, it differs from *H. pylori *having a wider metabolic flexibility in term of energy sources and electron transport chain. Moreover, *H. bizzozeronii *genome harbours higher number of MCPs compared to *H. pylori*, allowing the bacterium to respond to a wider spectrum of environmental signals. The high metabolic flexibility in combination with extraordinary genome plasticity gives to *H. bizzozeronii *the ability to easily move through several environments and adapt to different hosts. This ability is probably a common feature among all the species belong to *H. heilmannii *s.l. since both *H. felis *and *H. suis *genomes harbours a similar repertoire of genes involved in metabolism and chemotaxis observed in *H. bizzozeronii*. Thus, is tempting to speculate that the zoonotic nature of *H. heilmannii *s.l. infection in humans is explicable by the high metabolic versatility of these species which probably supports the growth of the microorganisms in a wide range of niches.

Although *H. bizzozeronii *lacks orthologues of some major *H. pylori *virulence genes, other candidate virulence factors are present. In particular, we identify a polysaccharide lyase as a potential new virulence factor of *H. bizzozeronii*. Efforts are ongoing to define molecular methods for *H. bizzozeronii *in order to provide experimental evidence of the function of potential new virulence factors involved in the developing of human gastritis and MALT lymphoma.

## Methods

### Cultivation, growth conditions and DNA extraction

The human *H. bizzozeronii *CIII-1 strain was isolated in March 2008 from a 45-year-old Finnish female who was suffering from severe dyspeptic symptoms and for whom antrum and corpus biopsies revealed chronic active gastritis [8]. The strain was cultured on HP medium (LabM Limited, Lancashire, UK) containing 5% of bovine blood and *Campylobacter *selective supplement (Skirrow, Oxoid Ltd., Cambridge, UK) for 4 days at 37°C in an incubator with a microaerobic atmosphere of 10% CO_2 _and 5% O_2 _(Thermo Forma, Series II Water Jacketed Incubator; Thermo Fisher Scientific, Waltham, MA 02454 USA). The high molecular-weight genomic DNA of *H. bizzozeronii *was extracted as described before [8], and the genomic DNA for the Solexa 5 kb mate pair library was extracted using a ZR Fungal/Bacterial RNA MiniPrep kit (Zymo Research Co, Irvine, CA, USA).

### Genome sequencing and assembly

The genome sequence of *H. bizzozeronii *CIII-1 was obtained as described previously [17]. Briefly, a combination of 454 Titanium (43x genome coverage, 8 kb mate pair library, performed by LGC Genomics GmbH, Berlin, Germany) and Solexa (50 cycles, 132x coverage, 5 kb mate pair library, performed by BaseClear BV, Leiden, The Netherlands) was assembled into 2 scaffolds representing a circular chromosome and a circular plasmid using the MIRA 3.2.1, SSAKE and the Staden software package. The assembly of the chromosome was validated by mapping to a *Mlu*I optical map produced by OpGen Inc. (Gaithersburg, Maryland, USA). The sequences were deposited in the EMBL bank under the accession numbers FR871757 and FR871758.

### Annotation and comparative genomic

For gene finding and automatic annotation, the sequences were uploaded to the RAST server [77]. We further analysed the coding sequences using the Artemis tool [78] and manually re-annotated the genes of special interest. The homologues were identified using NCBI's BLAST suite of programs with NCBI nr and nt and Swissprot as reference databases. Two proteins with amino acid identity greater than 40% over 80% sequence length were considered homologues. The conserved functional domains in proteins were identified using the hmmer program on Pfam database [79] as well as the InterProScan [80]. For the prediction of glycosyltransferases, glycoside hydrolases, polysaccharide lyases and carbohydrate esterases, we referred to the annotation available in the CAZy database [48]. The metabolic pathways were reconstructed using KEGG and SEED as reference databases [77,81]. The subcellular locations of proteins were predicted using the PSORTb tool via the psort web server [82], and signal peptides were identified using SignalP [55], TatP [83] and LipoP [84].

To identify the proteins unique to *H. bizzozeronii *as well as the proteins found in *H. pylori *but missing in *H. bizzozeronii*, we determined the core genome of 10 *H. pylori *strains (J99, 26695, B8, HPAG1, B38, P12, G27, Shi470, SJM180 and PeCan4) using OrthoMCL with a BLAST E-value cut-off of 1.0 e^-6 ^and an inflation parameter 1.5 [85]. We compared the core genome with the *H. bizzozeronii *CIII-1 genome, as previously described [86]. Briefly, based on the all-against-all BLASTp results, the proteins were clustered into groups of orthologues and recent paralogues. The proteins from *H. bizzozeronii *CIII-1 that did not cluster with any *H. pylori *proteins were considered to be specific to the species.

We identified the putative outer membrane proteins using BLASTp against the OMP database [87] and a set of known and categorised OMPs from *H. pylori *[42] using a BLASTp score ratio cut-off of 0.4 [88]. For further classification the resulting set of proteins was combined with the set of *H. pylori *OMPs and the phylogenetic relationships were analysed as described previously [13].

The *H. bizzozeronii *CIII-1 chromosome was searched for simple sequence repeats (SSRs) potentially involved in slipped-strand misparing using Msatfinder [89]. The thresholds for this study were as follows: mono (repeat unit length), > 8 (threshold value of repeat units); di > 5; tetra, penta, hexa and hepta, > 3. The positions of each of the SSRs (intergenic or intragenic) were identified using Artemis [78]. The lengths of all of the intragenic SSRs were artificially modified to assess the likely significance of the repeat-length variation on the expression of the associated reading frame.

The regions in the genome possibly acquired by horizontal gene transfer events as well as genomic islands were predicted using Alien Hunter [46] and Island Viewer [90], respectively. The potential prophage loci within the genome were predicted using the Prophage finder [76]. The possible IS elements were identified using the semi-automatic annotation system provided by ISsaga and were manually checked [70].

## Abbreviations

ACC: acetophenone carboxylase; AHD: allophanate hydrolase; NRS: nitrite reductase system; SNO: S- and N- oxidases; FDH: formate reductase system; GDH: glycerol-3-phosphate deydrogenase; PL: polysaccharide lyase; HYD: hydrogenase; NDH: NAD(P)H dehydrogenase; SDH/FR: succinate dehydrogenase/fumarate reductase; MQR: malate-quinone-reductase; LDH: lactate dehydrogenase; GLC: glycol oxidase; NAP: periplasmic nitrate reductase; MCP: methyl-accepting chemotaxis protein; CAC: citric acid cycle; PEP: phosphoenolpyruvate; TMAO: trimethylamine-n-oxide; DMSO: dimethyl sulfoxide; OMP: outer membrane protein; LPS: lipopolysaccharide; GT: glycosyltransferase; Tat: twin-arginine secretion system; MMR: mismatch repair system; SSR: simple sequence repeat; HGT: horizontal gene transfer; IS: insertion sequence; GI: genomic island; s.l.: sensu lato; s.s.: sensu stricto.

## Authors' contributions

TS performed the draft genome assembly and genome assembly validation and was responsible for the comparative genome analysis. PKK contributed to the data acquisition. MLH supervised the work at DFHEH. MR was responsible for the study design and coordination, contributed to the comparative genome analysis and wrote the manuscript. All authors read and approved the final manuscript

## Supplementary Material

Additional file 1 **supplementary tables**. Table S1, *Helicobacter bizzozeronii *CIII-1 methyl-accepting chemotaxis protein; Table S2, *Helicobacter bizzozeronii *CIII-1 CDS involved in metabolisms and respiration; Table S3, Outer Membrane Proteins of *Helicobacter bizzozeronii *CIII-1; Table S4, Glycosyltransferases and genes involved in the biosynthesis of glycans in *Helicobacter bizzozeronii *CIII-1; Table S5, ComB system in *Helicobacter bizzozeronii *CIII-1; Table S6, List the CDS belonging to *Helicobacter bizzozeronii *CIII-1 genomic island; Table S7, List the CDS belonging to *Helicobacter bizzozeronii *CIII-1 plasmid pHBZ1. Click here for file
